# Highly potent antimicrobial peptides from N-terminal membrane-binding region of *E. coli* MreB

**DOI:** 10.1038/srep42994

**Published:** 2017-02-23

**Authors:** Karabi Saikia, Yalavarthi Durga Sravani, Vibin Ramakrishnan, Nitin Chaudhary

**Affiliations:** 1 Department of Biosciences and Bioengineering Indian Institute of Technology Guwahati, Guwahati – 781 039, India

## Abstract

Microbial pathogenesis is a serious health concern. The threat escalates as the existing conventional antimicrobials are losing their efficacy against the evolving pathogens. Peptides hold promise to be developed into next-generation antibiotics. Antimicrobial peptides adopt amphipathic structures that could selectively bind to and disrupt the microbial membranes. Interaction of proteins with membranes is central to all living systems and we reasoned that the membrane-binding domains in microbial proteins could be developed into efficient antimicrobials. This is an interesting approach as self-like sequences could elude the microbial strategies of degrading the antimicrobial peptides, one of the mechanisms of showing resistance to antimicrobials. We selected the 9-residue-long membrane-binding region of *E. coli* MreB protein. The 9-residue peptide (C-terminal amide) and its N-terminal acetylated analog displayed broad-spectrum activity, killing Gram-negative bacteria, Gram-positive bacteria, and fungi. Extension with a tryptophan residue at the N-terminus drastically improved the activity of the peptides with lethal concentrations ≤10 μM against all the organisms tested. The tryptophan-extended peptides caused complete killing of *C. albicans* as well as gentamicin and methicillin resistant *S. aureus* at 5 μM concentration. Lipid-binding studies and electron microscopic analyses of the peptide-treated microbes suggest membrane disruption as the mechanism of killing.

Abuse of antibiotics has led to an alarming situation wherein many of the clinical strains have developed resistance against multiple antibiotics available. Emerging resistance to the existing antibiotics together with very slow discovery of new class of antibiotics is one of the major health threats today. Higher organisms have cohabited with microorganisms on this planet throughout their evolution and have devised strategies to control and combat them. Antimicrobial peptides (AMPs) happen to be one of the key members of such defense arsenal and play an important role to ward off the pathogens^1^. AMPs occur naturally in almost all organisms as an important element of their innate immune system^2^. AMPs have been exposed to microbes for millions of years but resistance against them is not prevalent. Moreover, during the course of evolution, mutations in the microbes have led to the diversification of AMPs^1^. The sequence diversity of the AMPs is such that classifying them based on their sequences is neither practical nor useful. Such diversity in AMPs could account for the inability of the microbes to develop good resistance against them thereby helping such an ancient weapon flourish throughout evolution. The activity of peptides could be altered by making subtle changes in their amino acid sequence, composition, and conformation thereby suggesting that the AMPs hold the promise to be developed into the next-generation antibiotics^3^.

Cationic antimicrobial peptides (CAMPs) i.e. the peptides possessing a net positive charge at neutral pH, constitute the largest group of the antimicrobial peptides^4^. Structurally, these peptides could attain diverse conformations such as α-helices, β-sheets, mixed conformations, loops, and extended structures^5^. An important feature of AMPs is that they fold into amphipathic structures that could interact with bacterial membranes^6^. Amphipathic α-helical peptides constitute the largest structural class among all the known CAMPs^7^. It is imperative to design antimicrobials that are cost effective and possess broad spectrum antimicrobial activity without instigating any toxic effect to the host. Naturally occurring antimicrobial peptides are often 20–50 residues long and high cost of peptide synthesis is one of the major obstacles in developing peptides as antibiotics^8^,^9^. The focus, therefore, is shifting towards designing and developing shorter antimicrobial peptides^10^.

MreB is a bacterial cytoskeleton protein present in non-spherical cells localized beneath the cell membrane in the form of filaments^11^. MreB attributes to the survival of non-spherical bacteria by maintaining the cellular structure akin to the cytoskeletal elements like actin, tubulin, and intermediate filaments present in eukaryotic cells. MreB proteins exist in two different forms, one with a short (~7–9) amino acid stretch at N-terminus that could fold into an amphipathic α-helix and the other that lacks this N-terminal region^11^. The N-terminal amphipathic helix in *E. coli* MreB is both necessary and sufficient for its membrane binding. We predicted that this N-terminal stretch could be developed into a potent antimicrobial peptide. Development of an amino acid sequence of microbial origin into an antimicrobial peptide is an interesting strategy as microbes should find it difficult to counter self-like sequences through conventional membrane modifications that are effected by resistant bacteria. Furthermore, the peptide could interfere with the native peptide stretches thereby disturbing the natural microbial processes.

## Results

### Peptides

The 9-residue peptide stretch (MLKKFRGMF) from *E. coli* was selected and four peptides were designed (Table 1).

Mean hydrophobicity (〈H〉) and mean hydrophobic helical moments (〈μH〉) were calculated using Heliquest web server^12^. A discrimination factor (D) is defined as 0.944 〈μH〉 +0.33(z) to identify the possible lipid-binding helices^12^,^13^. If the discrimination factor is higher than 1.34, the helix is predicted to be a membrane-binding one. As per the Heliquest discrimination factor criteria, all the four peptides qualified to be the lipid-binding ones. The peptides were synthesized, purified, and their identities ascertained using MALDI mass spectrometry (see Supplementary Figs S1 and S2).

### Surface activity and membrane binding of the peptides

**Figure 1:**
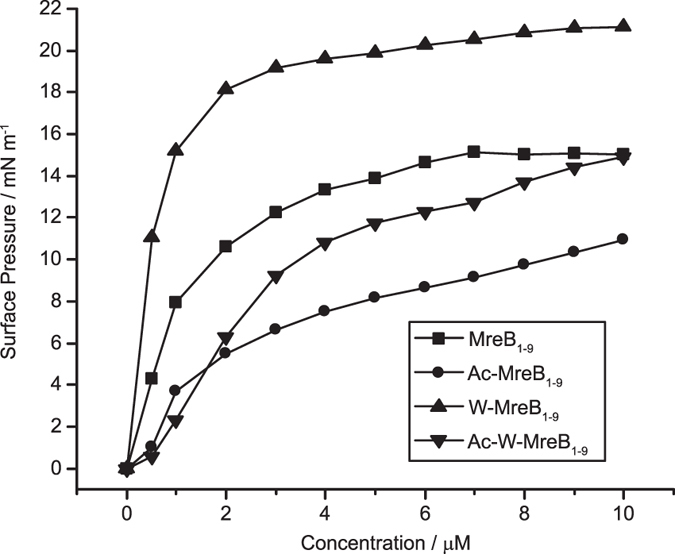
Surface activity of the peptides. Peptides at concentrations ranging from 0.5–10 μM were injected into the subphase. The stabilized surface pressure values are plotted against the peptide concentration.

Surface activity of the peptides was measured using 10 mM phosphate buffer, pH 7.4 as the aqueous subphase. Peptides were injected into the subphase and changes in the surface pressure were monitored by the Wilhelmy method using a platinum plate. All the peptides caused substantial enhancement in the surface pressure (≥5 mN m^−1^) even at 2 μM peptide concentration (Fig. 1).

The change in surface pressure, by and large, is higher for the peptides with higher net charge. Among the peptides with identical net charge, the longer peptides displayed larger changes in the surface pressure. Interaction of peptides with lipids was studied using lipid monolayers. 1-palmitoyl-2-oleoyl-*sn*-glycero-3-phosphocholine (POPC) and 1-palmitoyl-2-oleoyl-*sn*-glycero-3-phosphoglycerol (POPG) were used as the zwitterionic and negatively-charged lipids, respectively. Lipid monolayers of POPC and 7:3 POPC:POPG were prepared so as to obtain the surface pressures ~30–32 mN m^−1^. Without disturbing the lipid monolayers, the peptides were injected into the subphase so as to have 10 μM concentration. The peptides caused ~2–6 mN m^−1^ increase in the surface pressure for the POPC monolayer (Table 2). Ac-W-MreB_1–9_ caused 5.8 mN m^−1^ increase in the surface pressure while ≤3.6 mN m^−1^ change was observed for the other three peptides. With POPC:POPG monolayers, all four peptides caused large enhancement in the surface pressure. Ac-W-MreB_1–9_ caused 10 mN m^−1^ increase in surface pressure compared to 5.8 mN m^−1^ increase caused to POPC monolayers. Other three peptides caused ~8.5 mN m^−1^ increase to POPC:POPG surface pressure compared to ≤3.6 mN m^−1^ increase caused to POPC monolayers.

As predicted, all the peptides turn out to be the membrane-binding ones. The data clearly suggest that the peptides preferentially bind to the negatively-charged lipids as expected from the cationic, amphipathic peptides.

### Antimicrobial activity

Antimicrobial activity of the peptides was investigated against Gram-positive and Gram-negative bacteria as well as fungus. The minimal lethal concentrations of the peptides are shown in Table 3.

MreB_1–9_, the native sequence, possesses a net charge of +4 and exhibit high antimicrobial activity against both Gram-positive and Gram-negative bacteria as well as *C. albicans*. All the microorganisms tested, other than *S. aureus*, could be efficiently killed at 20 μM or lesser peptide concentration. The lethal concentration for *S. aureus* was 50 μM. Gentamicin-methicillin-resistant *S. aureus* (gentamicin-resistant MRSA), however, appeared more susceptible (lethal MreB_1–9_ concentration = 20 μM). Capping of N-terminus with acetyl group renders the peptide less effective, particularly against *P. aeruginosa* and *S. aureus*. Interestingly, the activity against *C. albicans* and gentamicin-resistant MRSA is not affected on N-terminal acetylation. Extension of MreB_1–9_ by a tryptophan residue at N-terminus makes the peptide (W-MreB_1–9_) at least 3 times more active. All the microbes tested were efficiently killed at ≤10 μM peptide concentration. Bacterial membranes are negatively charged and most cationic amphipathic antimicrobial peptides kill the bacteria by disrupting their membranes. A decrease in cationicity would adversely affect the peptide binding to microbial membranes thereby compromising their activity.

**Figure 2:**
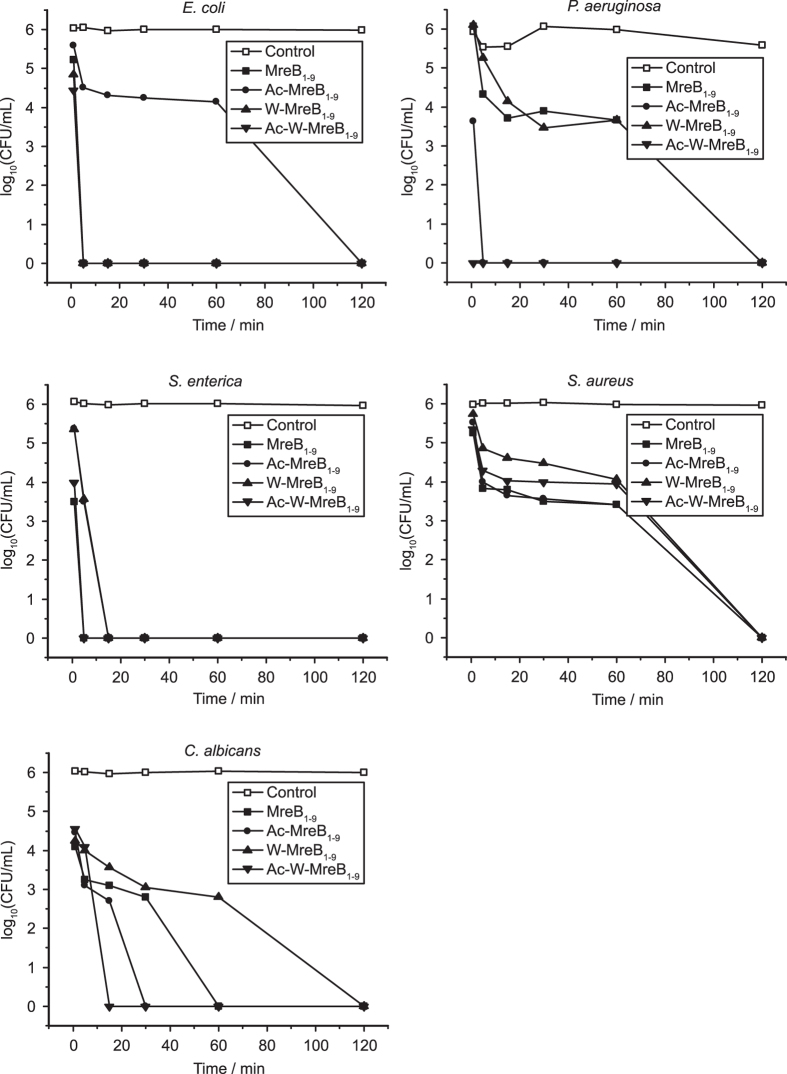
Kinetics of microbial killing by MreB-derived peptides. Microbes were treated with the peptides at their minimum lethal concentrations. Aliquots were taken out at different time points and antimicrobial activity was assayed as described in the ‘Materials and methods’ section.

The kinetics of killing in the presence of the lethal concentration of the peptides was examined (Fig. 2). The peptides exhibited strain-dependent killing-kinetics. All the four peptides caused rapid killing of *S. enterica*; the bacteria was completely killed within 15 minutes of peptide treatment. *S. aureus*, on the other hand, showed >50% survival even 1 h after the peptide treatment. The bacteria, however, was completely killed in 2 hours. Ac-W-MreB_1–9_ displayed the most rapid killing against *E. coli, P. aeruginosa, S. enterica*, and *C. albicans*. All the three bacteria were completely killed within 5 minutes of peptide treatment while *C. albicans* was killed within 15 minutes. Killing of *S. aureus* was slower and all the peptides followed very similar kinetics. The kinetics of killing are comparable or better than several established antimicrobial peptides^14^,^15^.

**Figure 3:**
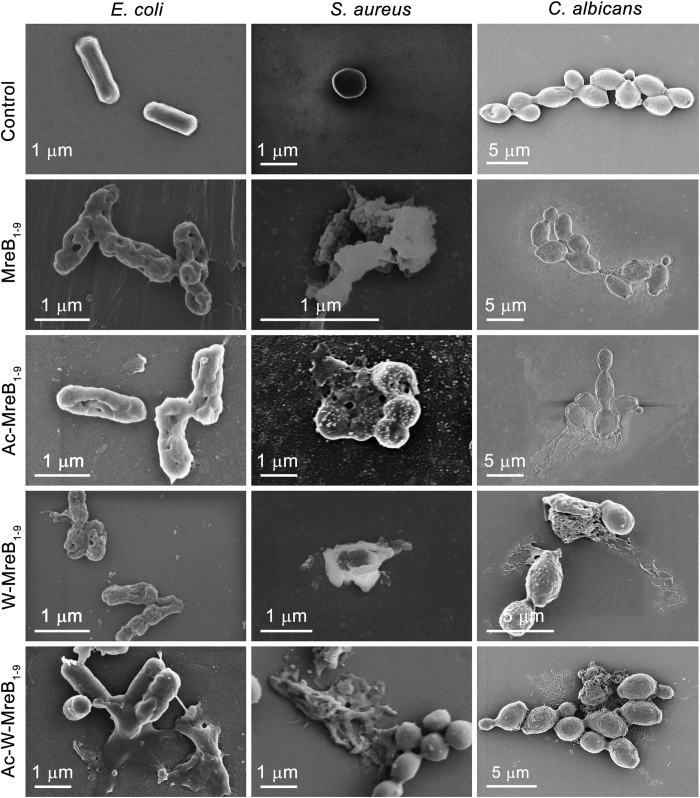
FESEM images of the peptide-treated *E. coli, S. aureus*, and *C. albicans.* Microbes were treated with the peptides at their minimum lethal concentrations and incubated at 37 °C (bacteria) and 28 °C (fungus) for 2 hours. The cells were harvested, fixed, and analysed using FESEM as described in the ‘Materials and methods’ section.

Field emission scanning electron microscopic (FESEM) images of the peptide-treated microbes are shown in Fig. 3. The peptide-treated microbes displayed unusual morphology. Treatment with the peptides caused pore formation in the bacterial membrane alongside large-scale perturbation. Similar membrane perturbation was observed for *C. albicans* as well. The microscopic analysis suggests membrane perturbation as the mechanism of killing.

### Salt sensitivity

The peptides retained substantial activity in the presence of 100 mM NaCl (Table 4). All the peptides displayed >75% killing of all the bacteria at their lethal concentrations. The activity against *C. albicans*, however, reduced to 38–54%.

Antibacterial activity of Ac-W-MreB_1–9_ was little affected by the presence of salt showing complete killing of *E. coli, P. aeruginosa*, and *S. enterica;* and >90% killing of *S. aureus*. Activity against *C. albicans*, however reduced to ~43%. Divalent cations showed varied affects on the activity of the peptides. MreB_1–9_ retained >50% activity against all the microorganisms tested. Activity of Ac-MreB_1–9_, on the other hand, was severely compromised against *E. coli* and *S. enterica* in the presence of divalent cations. Both MreB_1–9_ and Ac-MreB_1–9_ retained >90% activity against *S. aureus* in the presence of salt as well as divalent cations. Tryptophan-extended peptides exhibited better activity than the native sequences. The activity of W-MreB_1–9_ against *E. coli* and *P. aeruginosa*, however, got severely compromised in the presence of Mg^2+^ ions. Ac-W-MreB_1–9_, on the other hand, retained ~80% or more activity against all the four bacteria as well as fungus in the presence of divalent cations. MreB_1–9_ is the sequence derived from *E. coli* and it is interesting to see that the native MreB peptide, MreB_1–9_ retains >50% activity against *E. coli* in the presence of salt and divalent cations. The varied response to the divalent cations suggests that the peptides could work synergistically to achieve broad spectrum killing under physiological conditions.

### Hemolytic assay

Lysis of human erythrocytes by the peptides was examined at 50 and 100 μM peptide concentrations (Table 5). No hemolysis was caused by MreB_1–9_, Ac-MreB_1–9_, and W-MreB_1–9_ at 50 μM concentration while ~4% hemolysis was caused by Ac-W-MreB_1–9_. The lethal concentrations of W-MreB_1–9_ and Ac-W-MreB_1–9_ are ≤10 μM against all the organisms tested (Table 3). W-MreB_1–9_ did not cause any hemolysis while Ac-W-MreB_1–9_ caused <5% hemolysis at 5-fold higher concentrations. W-MreB_1–9_ caused little hemolysis (1.62%) even at 10-fold higher concentration.

## Discussion

Emergence of multidrug-resistant microbes demands for the development of new class of antimicrobials and peptides are being looked at with high expectations^16^. Antimicrobial peptides are natural defense molecules found in all life forms. They have sustained the evolutionary pressure without causing any significant antimicrobial resistance making them the molecules of choice for developing the new generation of antimicrobials.

We realized the antimicrobial potential of the 9-residue long N-terminal amphipathic helix of *E. coli* MreB protein and designed four peptides. The peptides displayed broad spectrum activity, killing Gram-negative bacteria (*E. coli, P. aeruginosa, S. enterica*), Gram-positive bacteria (*S. aureus* and gentamicin-resistant MRSA), and fungus (*C. albicans*). The lack of discovery of the new antibiotics against Gram-negative bacteria is alarming. The new antibiotics that entered medicine in last five decades were all against the Gram-positive bacteria. Largely due to the lack of new antibiotics that could combat Gram-negative bacteria, Gram-negative bacteremia has become a serious health issue^9^,^17^,^18^. Among Gram-negative bacteria, *P. aeruginosa* turns out to be one of the most common human pathogens. It is one of the two most common pathogens causing nosocomial pneumonia, *S. aureus* being the other one. *P. aeruginosa* is an opportunistic pathogen and the serious Pseudomonal infections are often hospital acquired^19^. It happens to be the most commonly isolated pathogen from patients that are hospitalized for long durations especially the ones that have compromised immune system. *P. aeruginosa* is life-threatening for the critically ill patients in the intensive care units. It is an intrinsically resistant bacterium to many antibiotics and is capable of acquiring resistance to multiple antibiotics^17^,^19^. *E. coli* and *S. enterica* are other clinically-relevant Gram-negative bacteria. All three bacteria were killed by the native sequence, MreB_1–9_ at lethal concentrations comparable to many established antimicrobial peptides^20^. Extension of MreB_1–9_ at the N-terminus by a tryptophan residue improved the activities drastically. All three Gram-negative bacteria could be completely killed at 5 μM or lower peptide concentration, an activity comparable or better than many highly-active antimicrobial peptides^20^. Indolicidin, for example, displays an MIC of 67 μM against *P. aeruginosa, S. aureus*, and *C. albicans*; the MIC against *E. coli* was >134 μM. Human cathelicidin, LL-37 displays an MIC >50 μM against *E. coli, P. aeruginosa, S. aureus*, and *C. albicans*. Melittin, the highly lytic peptide from honey bee venom displays an MIC of 2.8 μM against *S. aureus*, 11.2 μM against *C. albicans*, and 22.4 μM against *E. coli* and *P. aeruginosa*. Cecropin A, an insect antimicrobial peptide, displays lethal concentrations of 0.32 μM against *E. coli* and 3.5 μM against *P. aeruginosa*^21^. PR-39, an AMP from pig intestine displays lethal concentrations of 0.3 μM against *E. coli* and 200 μM against *P. aeruginosa* and *S. aureus*^22^. The activity of the peptides was antagonised by the salt and divalent cations in a species-dependent manner. The outer membrane of Gram-negative bacteria contain lipopolysaccharide (LPS) as one of the major molecular components of the outer leaflet. The structure of LPS is characterized by a variable number of phosphate groups and anionic sugars in the oligosachharide core. The negatively charged groups are bridged by divalent cations^1^,^23^. The antimicrobial peptides bind the LPS by displacing the divalent cations. The structural diversity of the LPS contribute to the differential susceptibility of the Gram-negative bacteria to the antimicrobial peptides. There are many threatening Gram-positive bacteria, *S. aureus* being one of the most clinically-relevant ones. *S. aureus* is the most common bacterium present on human skin. It is considered an opportunistic pathogen but more aggressive strains have also evolved. *S. aureus* infections could be easily treated using Penicillin in 1940s^24^. Subsequently, *S. aureus* developed resistance against penicillin and could be treated with a Penicillin analog, Methicillin. A methicillin-resistant *S. aureus* strain was identified in 1960s and this was the birth of difficult to treat methicillin-resistant *S. aureus* (MRSA)^25^. It is arguably the most important hospital-acquired human pathogen. Although historically a hospital-acquired infection, community-acquired MRSA strains have emerged and are now epidemic in United States. It is very urgent to come up with new antimicrobials to treat MRSA infections. MreB_1–9_ and its acetylated analog displayed lower activity against *S. aureus* compared to that against the Gram-negative bacteria. However, MRSA could be efficiently killed at concentrations comparable to those required for killing Gram-negative bacteria. N-terminal extension with a tryptophan residue drastically improves the activity wherein both methicillin susceptible and resistant bacteria could be killed at ≤10 μM peptide concentrations. The most notable feature is that all the peptides retained >70% activity against *S. aureus* in the presence of salt and divalent cations. The peptides displayed excellent antifungal activity as well, killing *C. albicans* at concentrations as low as 5 μM. Candida is a clinically-important fungus and *C. albicans* happens to be the most common species causing invasive candidiasis^26^. All the four peptides could efficiently kill *C. albicans* and retained substantial activity in the presence of salt and divalent cations.

## Conclusion

Interaction of proteins with membranes is an indispensable process for any life form and microbes are no exception. This study reports that the lipid-binding stretch of *E. coli* MreB could be developed into a potent, wide-spectrum antibiotic, killing both Gram-negative and Gram-positive bacteria as well as yeast. Development of the membrane-perturbing antimicrobial peptides from the membrane-binding stretches of microbial proteins, therefore, could be an excellent strategy to combat microbes especially the antibiotic-resistant ones. A self-like sequence would be less prone to enzymatic degradation by the bacteria. Furthermore, as these peptides kill the microbes by permeating/perturbing the cell membranes rather than targeting an intracellular target, they are less likely to induce resistance in the pathogens.

## Materials and Methods

### Materials

NovaPEG Rink amide resin, Fmoc-protected amino acids, and *N,N,N’,N’*-tetramethyl-*O*-(1*H*-benzotriazol-1-yl)uronium hexafluorophosphate (HBTU) were purchased from Novabiochem (Darmstadt, Germany). *N,N*-diisopropylethylamine, trifluoroacetic acid, ethanedithiol, thioanisole, piperidine, and acetic anhydride were from Sigma-Aldrich Chemicals Pvt. Ltd. 1-hydroxybenzotriazole hydrate, diethyl ether, and ethylenediaminetetraacetic acid disodium salt were from Sisco Research Laboratory, India. *N,N*-dimethylformamide and *m*-cresol were purchased from Merck India. Lipids were obtained from Avanti Polar Lipids. Media and supplements for growing bacteria and fungi were obtained from HiMedia. The bacterial strains used were *Escherichia coli* (MG 1655), *Pseudomonas aeruginosa* (NCTC 6750), *Salmonella enterica* (SL 4213), *Staphylococcus aureus* (NCTC 8530), and Gentamicin and Methicillin resistant *Staphylococcus aureus* (ATCC 33592) while *Candida albicans* (ATCC 18804) was used for the antifungal assay.

### Peptides synthesis

Peptides were synthesized manually by solid-phase peptide synthesis using Fmoc chemistry. N-terminal acetylation was carried out on-resin using 5 equivalents of acetic anhydride and 10 equivalents of *N,N*-diisopropylethylamine. The peptides were cleaved from the resin using a cleavage cocktail comprising trifluoroacetic acid, ethanedithiol, thioanisole, and *m*-cresol (20:1:2:2). The peptides were precipitated in ice-cold diethyl ether and purified on Shimadzu Prominence Modular HPLC instrument on a reversed-phase C18 column using a linear gradient of water and acetonitrile (10–100%) containing 0.1% TFA. The purity of all the peptides was >95% as ascertained by analytical reversed-phase HPLC (see Supplementary Fig. S1). The peptides were characterized using matrix-assisted laser desorption/ionization mass spectrometry on a Bruker, Autoflex Speed MALDI TOF/TOF (see Supplementary Fig. S2). The stock solutions of 0.5–1 mM were prepared in deionized water and concentrations estimated using a molar absorption coefficient of 5690 M^−1^ cm^−1^ at 280 nm for tryptophan-containing peptides and a molar absorption coefficient of 286 M^−1^ cm^−1^ at 254 nm for the tryptophan-lacking peptides.

### Surface activity and membrane binding

Surface activity and membrane binding studies were carried out on a KSV Nima Langmuir instrument (Biolin Scientific) using a custom made polytetrafluoroethylene trough of 13.2 cm^2^ area. Surface pressure was measured by Wilhelmy method using a platinum plate (Biolin Scientific). Phosphate buffer (10 mM, pH 7.4) was used as the aqueous subphase. Peptides were injected into the subphase and increase in surface pressure was monitored over time. The maximal surface pressure change was plotted against the peptide concentration^27^.

Lipids dissolved in chloroform were spread on the subphase to achieve initial surface pressure of 30–32 mN m^−1^. The peptides were injected into the subphase to obtain a concentration of 10 μM and mixing was achieved by magnetic stirring. The increase in surface pressure caused by the peptides was recorded as a function of time. The maximal changes in the surface pressure are reported.

### Antimicrobial assay

Mid-log phase cells were harvested and washed twice with 10 mM phosphate buffer of pH 7.4. The cells were then diluted in the same buffer so as to have approximately 10^6^ colony forming units (CFU)/mL. One hundred microliter of the cell suspensions were treated with different concentrations of the peptides (final volume adjusted to 120 μL) and incubated at 37 °C (bacteria) and 28 °C (fungus) for 2 hours. Following incubation, the cell suspensions were 10-fold diluted in 10 mM phosphate buffer, pH 7.4 and 20 μL volumes were spread on nutrient agar plates (bacteria) and yeast extract-peptone-dextrose (YPD) agar plates (fungus). Bacterial plates were incubated at 37 °C for 12–18 hours while fungal plates were incubated at 28 °C for 24–30 hours. The colonies were counted to determine the antimicrobial activity. The peptide concentrations that resulted in complete killing of the microbes were considered the minimum lethal concentrations.

### Killing kinetics

The microbes were treated with the minimum lethal concentration of the peptides. The killing kinetics assay was performed by taking out 20 μL aliquots of the peptide-treated microbes after 1, 5, 15, 30, 60, and 120 minutes. The activity was determined as mentioned in the “Antimicrobial assay” section.

### Salt sensitivity assay

Mid-log phase microbial cells were diluted in 10 mM phosphate buffer, pH 7.4 having 100 mM NaCl or 2 mM CaCl_2_ or 1 mM MgCl_2_. Peptides at their lethal concentrations were added to 100 μL microbial suspensions (final volume adjusted to 120 μL) and incubated at 37 °C (bacteria) and 28 °C (fungus) for 2 hours. Aliquots of 20 μL were 10-fold diluted and spread-plated. Colonies were counted to determine the activity.

### Hemolytic assay

Human blood (~2 mL) was collected from a healthy individual in a tube containing ethylenediaminetetraacetic acid disodium salt and centrifuged at 800 × g for 5 minutes. The pellet was resuspended in 5 mM HEPES buffer, pH 7.4 containing 150 mM NaCl. Erythrocytes were washed several times and a 5% hematocrit was prepared in the same buffered saline. Peptides at 50 μM and 100 μM concentrations were incubated with 100 μL of 5% hematocrit and incubated for 1 hour at 37 °C. Following incubation, the hematocrit was centrifuged at 800 × g for 5 minutes and the absorbance of the supernatant was recorded at 540 nm. Hematocrit incubated with deionized water was considered as the positive control (100% lysis).

### FESEM analysis

Mid-log phase cells (10^7^) were treated with the minimal lethal concentrations of the peptides and incubated at 37 °C (bacteria) and 28 °C (fungus) for 2 hours. Incubated cells were centrifuged at 5000 × g for 5 minutes. The pellets were treated with 2.5% glutaraldehyde and incubated at 4 °C for 3 hours. Subsequently, cells were centrifuged at 5000 × g for 5 minutes and the supernatant was discarded. Cells were washed twice with phosphate buffer, loaded on a glass coverslip and dried at room temperature. The loaded samples were washed with deionized water and ethanol gradient ranging from 30–100%. The samples were air-dried, sputter-coated with gold, and analysed using FESEM.

### Declaration

The experiments and methods associated with human blood were carried out in “accordance” with the relevant guidelines. All the experimental protocols were approved by the ethics committee of Indian Institute of Technology Guwahati. An informed consent was obtained from all subjects.

## Additional Information

**How to cite this article**: Saikia, K. *et al*. Highly potent antimicrobial peptides from N-terminal membrane-binding region of *E. coli* MreB. *Sci. Rep.* **7**, 42994; doi: 10.1038/srep42994 (2017).

**Publisher's note:** Springer Nature remains neutral with regard to jurisdictional claims in published maps and institutional affiliations.

## Supplementary Material

Supplementary Information

## Figures and Tables

**Table 1:** Sequences and physicochemical properties of the peptides.

Peptide name	Peptide sequence[a]	z[b]	〈H〉^[c]^	〈μH〉[c]	D
MreB_1–9_	MLKKFRGMF-am	+4	0.528	0.762	2.039
Ac-MreB_1–9_	Ac-MLKKFRGMF-am	+3	0.528	0.762	1.709
W-MreB_1–9_	WMLKKFRGMF-am	+4	0.7	0.523	1.814
Ac-W-MreB_1–9_	Ac-WMLKKFRGMF-am	+3	0.7	0.523	1.484

^[a]^‘Ac-’ at N-terminus represents acetylated amino-terminus, while ‘-am’ at C-terminus represents C-terminal amide.

^[b]^Net charge on the peptide at pH 7.4

^[c]^〈H〉 and 〈μH〉 were calculated using Heliquest web server^12^.

**Table 2:** Maximum increase obtained in the surface pressure of the lipid monolayers on peptide addition (10 μM initial subphase concentration).

Lipid monolayer	Increase in surface pressure (mN m^−1^)			
	MreB_1–9_	Ac-MreB_1–9_	W-MreB_1–9_	Ac-W-MreB_1–9_
POPC	3.6	1.8	3.6	5.8
POPC:POPG (7:3)	8.3	8.5	8.7	10

**Table 3:** Antimicrobial activity of the peptides.

Microbe	Minimum Lethal Concentration (μM)[a]			
	MreB_1–9_	Ac-MreB_1–9_	W-MreB_1–9_	Ac-W-MreB_1–9_
*Escherichia coli*	20	15	5	10
*Pseudomonas aeruginosa*	10	100	3	10
*Salmonella enterica*	10	15	3	2
*Staphylococcus aureus*	50	100	10	10
*Staphylococcus aureus (Gentamicin and Methicillin-resistant*)	20	20	5	5
*Candida albicans*	15	15	5	5

The assays were carried out with the mid-log phase cells in 10 mM phosphate buffer, pH 7.4. ^[a]^Minimum peptide concentration that resulted in complete killing of the bacteria/fungus.

**Table 4:** Antimicrobial activity of the MreB-derived peptides at their lethal concentrations in the presence of 100 mM NaCl, 2 mM CaCl_2_, and 1 mM MgCl_2_.

	Microbe →Peptide ↓		*E. coli*	*P. aeruginosa*	*S. enterica*	*S. aureus*	*Gentamicin-resistant MRSA*	*C. albicans*
Percentage killing	MreB_1–9_	NaCl	99.7 ± 0.4	90.0 ± 13.9	96.6 ± 2.7	98.9 ± 1.3	96.7 ± 2.7	38.1 ± 5.9
		MgCl_2_	53 ± 9.6	76.2 ± 8.1	79.1 ± 1.0	90.8 ± 9.2	98.8 ± 0.7	98.0 ± 1.6
		CaCl_2_	69.5 ± 1.4	99.7 ± 0.4	52.4 ± 10.8	98.0 ± 2.1	95.6 ± 4.1	99.6 ± 0.2
	Ac-MreB_1–9_	NaCl	98.6 ± 1.9	97.8 ± 1.4	78.6 ± 11.0	99.6 ± 0.6	93.2 ± 7.7	54.0 ± 9.6
		MgCl_2_	0	42.0 ± 5.8	65.6 ± 6.7	93.6 ± 9.2	97.2 ± 3.6	98.5 ± 1.0
		CaCl_2_	4.6 ± 3.4	100	2.5 ± 1.1	97.4 ± 3.9	96.4 ± 1.8	57.0 ± 6.1
	W-MreB_1–9_	NaCl	99.7 ± 0.4	93.0 ± 9.7	98.2 ± 0.1	87.6 ± 3.0	96.8 ± 3.5	45.7 ± 8.8
		MgCl_2_	0	15.1 ± 4.1	72.7 ± 9.4	91.7 ± 7.8	97.9 ± 2.7	96.8 ± 3.2
		CaCl_2_	10.4 ± 3.2	99.8 ± 0.1	99.5 ± 0.2	74.5 ± 2.9	95.8 ± 4.8	58.3 ± 12.0
	Ac-W-MreB_1–9_	NaCl	100	100	100	93.5 ± 9.7	92.0 ± 8.8	43.4 ± 8.9
		MgCl_2_	100	100	96.7 ± 3.8	79.9 ± 4.9	95.7 ± 3.0	99.9 ± 0.1
		CaCl_2_	100	100	100	83.5 ± 1.4	89.6 ± 3.3	92.5 ± 8.8

**Table 5:** Percentage lysis of human erythrocytes by the MreB-derived peptides.

Peptide concentration (μM)	Percentage hemolysis			
	MreB_1–9_	Ac- MreB_1–9_	W- MreB_1–9_	Ac-W- MreB_1–9_
50	0	0	0	4.15 ± 0.96
100	0	0	1.62 ± 0.08	11.88 ± 2.44
