# Supplementary material for: Highly potent antimicrobial peptides from N-terminal membrane-binding region of E. coli MreB
Source: Sci Rep. 2017 Feb 23;7:42994. doi: 10.1038/srep42994 (PMC5322399; doi:10.1038/srep42994)
Supplement: Supplementary Information [file srep42994-s1.doc]

**Supplementary Information**

Highly potent antimicrobial peptides from N-terminal membrane-binding region of *E. coli* MreB

Karabi Saikia, Yalavarthi Durga Sravani, Vibin Ramakrishnan and Nitin Chaudhary*

Department of Biosciences and Bioengineering

Indian Institute of Technology Guwahati, Guwahati – 781 039

INDIA

*Author for correspondence: Nitin Chaudhary

Tel: +91-361-2582224

Fax: +91-361-2582249

E-mail: [chaudhary@iitg.ernet.in](mailto:chaudhary@iitg.ernet.in)

**
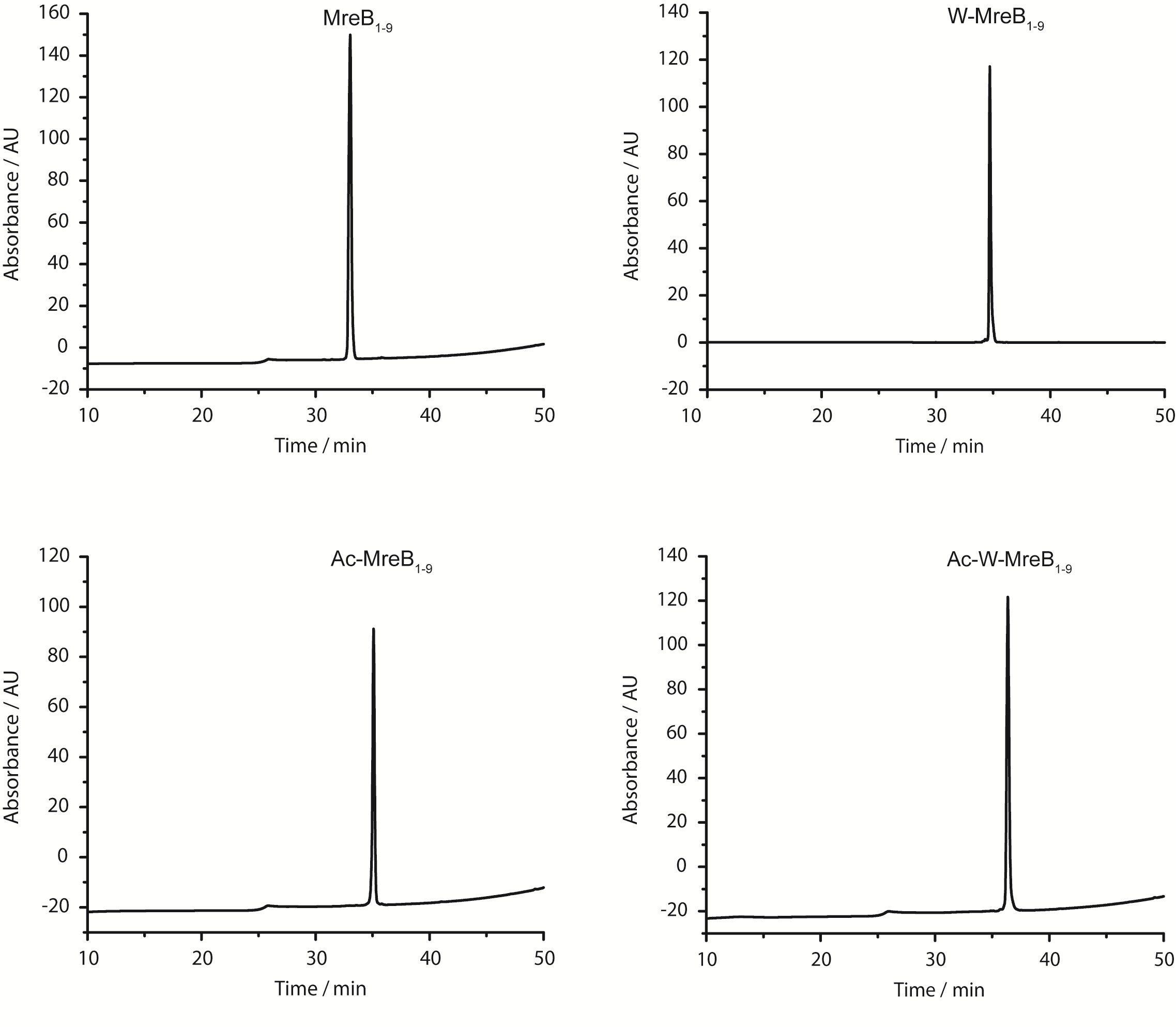
**

Fig. S1. Reversed-phase HPLC chromatograms of the purified MreB-derived peptides.

**
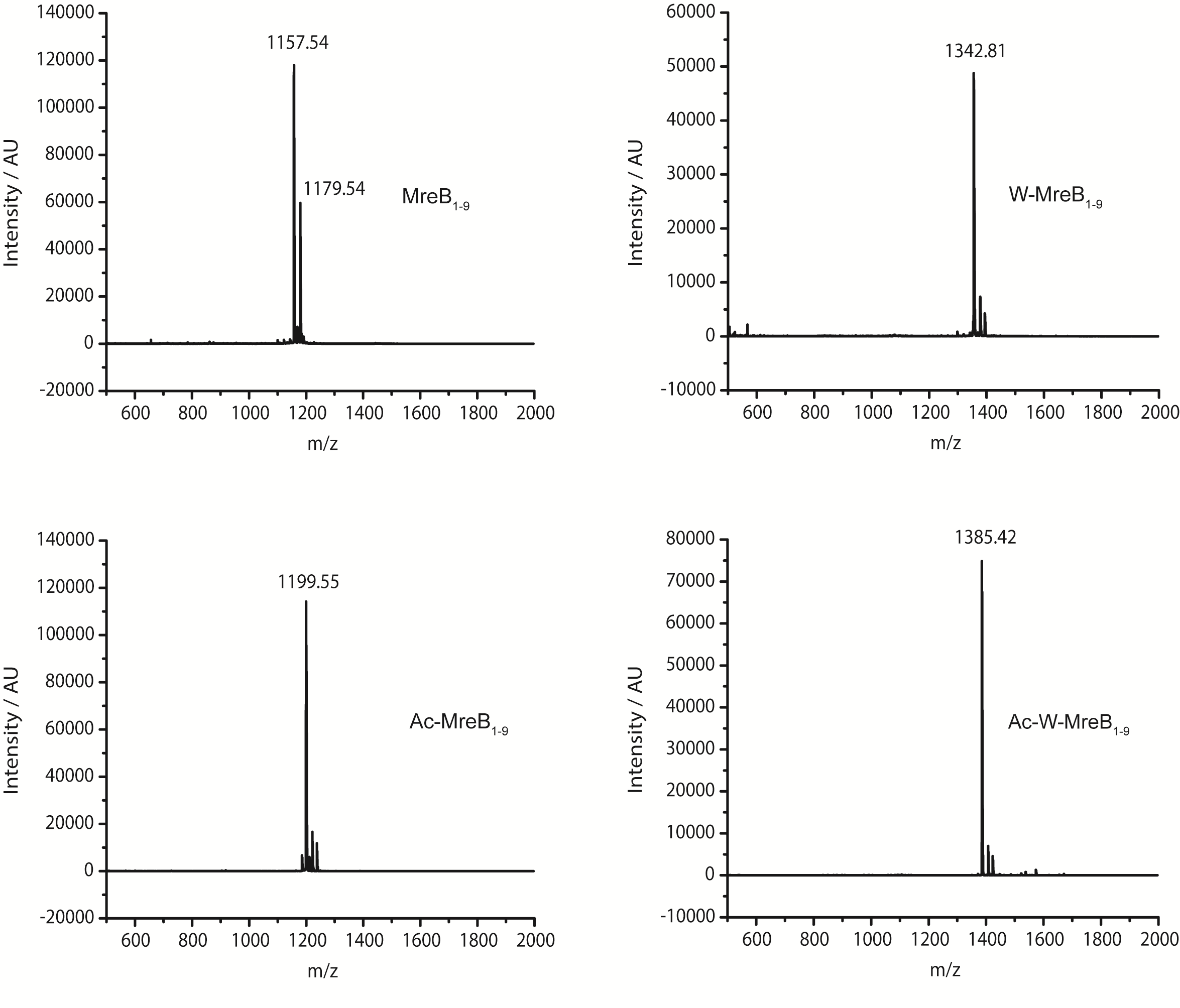
**

Fig. S2. MALDI mass spectra of the purified MreB-derived peptides. The calculated monoisotopic masses for MreB_1-9_, Ac-MreB_1-9_, W-MreB_1-9_, and Ac-W-MreB_1-9_ are 1155.63 Da, 1197.64 Da, 1341.71 Da, and 1383.72 Da, respectively. The additional peak in the MreB_1-9_ MALDI mass spectrum with m/z of 1179.54 Da corresponds to sodium adduct and does not signify any impurity in the peptide.
